# Stairs and Doors Recognition as Natural Landmarks Based on Clouds of 3D Edge-Points from RGB-D Sensors for Mobile Robot Localization†

**DOI:** 10.3390/s17081824

**Published:** 2017-08-08

**Authors:** Leonardo A. V. Souto, André Castro, Luiz Marcos Garcia Gonçalves, Tiago P. Nascimento

**Affiliations:** 1 Natalnet Labs, Universidade Federal do Rio Grande do Norte (UFRN), 59078-970 Natal, RN, Brazil; leonardoangelo8@gmail.com (L.A.V.S.); lmarcos@dca.ufrn.br (L.M.G.G.); 2 LaSER-Embedded Systems and Robotics Lab, Universidade Federal da Paraiba (UFPB), 58058-600 João Pessoa, PB, Brazil; andrelobo27@gmail.com

**Keywords:** natural landmarks, RGB-D sensors, 3D edge-point cloud, robot localization

## Abstract

Natural landmarks are the main features in the next step of the research in localization of mobile robot platforms. The identification and recognition of these landmarks are crucial to better localize a robot. To help solving this problem, this work proposes an approach for the identification and recognition of natural marks included in the environment using images from RGB-D (Red, Green, Blue, Depth) sensors. In the identification step, a structural analysis of the natural landmarks that are present in the environment is performed. The extraction of edge points of these landmarks is done using the 3D point cloud obtained from the RGB-D sensor. These edge points are smoothed through the $Sl_{0}$ algorithm, which minimizes the standard deviation of the normals at each point. Then, the second step of the proposed algorithm begins, which is the proper recognition of the natural landmarks. This recognition step is done as a real-time algorithm that extracts the points referring to the filtered edges and determines to which structure they belong to in the current scenario: stairs or doors. Finally, the geometrical characteristics that are intrinsic to the doors and stairs are identified. The approach proposed here has been validated with real robot experiments. The performed tests verify the efficacy of our proposed approach.

## 1. Introduction

An ability that is expected in autonomous robots is the capacity of collecting useful information from their surroundings. For that, in general, robots use sensors to acquire raw data and generate the useful information from the surrounding environment through some embedded computer processing. With this information provided, a robot can perform a large range of tasks including robot localization, which are algorithms that enable a robot to calculate its pose (position and orientation) in relation to an origin in a given map. This approach is one of the most studied mobile robotic problems [1]. One such approach to localization is the extraction and use of natural landmarks. The use of natural landmarks in mobile robot localization remains a challenge due to the differences in shape, design and the influence of illumination conditions. As an example, a simple algorithm for object recognition has a large number of false positives or false negatives in recognizing different types of doors and staircases in office buildings.

Therefore, we propose an approach for natural landmarks identification and recognition to be used in mobile robots localization. We use 3D edge clouds created from the data given by the above devices as the underlying structure. The proposed approach uses the edges of planes as a key characteristic of the natural landmark. We tested our approach in an indoor environment of a faculty building. On the recognition phase, we filter the resultant cloud and apply a recognition unsupervised algorithm that is also a proposal of this work, in order to retrieve intrinsic geometric characteristics regarding doors and stairs (or similar structures). The proposed algorithm is validated by way of real robot experiments, which demonstrate the performance of our method. The experiments were performed to demonstrate the algorithm efficacy in detecting the marks. Our approach achieves numbers as high as 96.5% in precision and 98.9% of accuracy.

Technological improvements with low-cost 3D sensors (e.g. RGB-D sensors) has enabled robots to perceive the environment as a 3D spatial structure. Note that more complex types of landmarks appear from this kind of data that includes, besides RGB (intensity), points in 3D world coordinates, thus increasing by one the dimension of data. Ambiguities and other problems such as illumination that occur using only 2D coordinates and RGB data can be solved here, however at a higher cost. The effectiveness of landmark identification and recognition is associated with the existence of marks in the environment, and also with sensor precision and frame rate, and with the capacity of the robot system processing. Nonetheless, a problem that remains is to define what type of low-level features can be used for landmark selection, mainly considering that several novel devices can provide more useful data, such as the Kinect cameras, ZED cameras, Bumble-bee cameras, and Minoru stereo cameras.

Several types of sensors are used for the robotic localization problem [2,3]. However, taking into account the relation between the sensor cost-reliability and measurement errors, camera-based sensors are, so far, the most accurate. They also have the advantage of being inexpensive and intuitive to human interaction. In addition, the visual sensors are able to recover environmental data, allowing the computation of particular information of the scenario that contributes to a more robust robot localization approach. One of the techniques used to localize robots is called a global view and it is approached by Lee et al. [4], Peipei et al. [5] and by Nascimento et al. [6]. In all of these approaches, ceiling mounted cameras provide an overview of the environment and the robotic platforms. Their approaches contain severe limitations regarding an environment that has corridors and rooms. Furthermore, several vision based localization techniques can be found in the literature [2,3,7,8]. The most used and researched localization algorithms (MCL [7], KF [8], EKF [3], etc.) aim to correct the errors accumulated over time coming from robot displacement.

Catadioptric cameras [9], fish-eye cameras [10], stereo cameras [11] or the more recent RGB-D devices [12] are widely used in mobile robotics. For landmark based localization, which can be either artificial or natural, the usual technique is done by means of identification and recognition of objects (landmarks) in the environment and posterior calculation of the robot’s pose with respect to the found object. Artificial landmarks such as Quick Response codes (QR-codes) have been being used recently in mobile robot landmark localization [13,14]. Specific designed patterns are also used in artificial landmark localization [15] in order to attend specific purposes or to overcome specific problems, such as the ones found in industry [16]. In contrast, natural landmarks are also used in mobile robot localization. The most common natural landmarks are lamps on the ceiling [17,18]. Finally, in natural landmark recognition, some authors use descriptors (SIFT [19] or SURF [12]) for acquiring characteristics from the environment.

Regarding natural landmarks, Liu et al. [20] propose an approach for the extraction of characteristics using the data captured by a 2D laser scanning. The algorithm extracts reference points (i.e., points relating to borders, curved segments of center, among others) that could be from a natural landmark. The process is basically done through a proposed curvature scale geometrical identification approach.

Furthermore, Rous et al. [21] propose an approach for mobile robot navigation based on the observation of natural landmarks contained in a certain scene using a monocular camera attached to the robot as the main sensor. The algorithm applies an analysis in the structures’ detection and chooses the most relevant one for the robot navigation task. However, this detection depends on prior knowledge about the structure that would be found. Examples of such structures would be doors or previously chosen objects. The process begins with a selective orientation based on the Hough-transform (OHT). This process generates a lattice of convex polygons. The homogeneous polygons are segmented and merged in the reference region, and finally an extraction is performed.

Xiong and Choi [22] correct the estimated robotic platform position based on the comparison between natural landmarks and some references assigned in the scene. The approach uses a fish-eye camera and the error correction process makes use of an estimation concerning the relationship between the natural landmark identification along with the distortion of the sensor calibration data. Another work by Haytham et al. [23] proposes a 2D mapping and localization approach applied to UAV. Such application has as its main objective to reduce the accumulated error from robot displacement. The authors present a method for real-time matching of 2D characteristics.

Finally, Chai et al. [24] introduce an approach to identify straight lines and homogeneous colors within the environment. From these particular environmental area features, the algorithm would be able to identify objects in order to use these objects as natural landmarks. Their work compares selected points using the Harris corner detector. They take as a guideline for the selection of natural landmarks a combination of straight lines to form convex polygons. Notice that Rous et al. [21], whose work is described above, make use of polygon features as well. In contrast, Chai et al. [24] also demonstrate a method for detecting lines in order to simplify the creation of polygons.

The main difference between the aforementioned works and the method that we propose in this paper is the generality of our approach. Since the identification stage has been introduced in our previous work [25], here we demonstrate that our recognition process is generic in the sense that it can recognize staircases and doors independent of the robot pose and the state of the object (closed door, opened door, semi-opened door). More importantly, the recognition step is performed in real-time without the need of an offline training step and without jeopardizing the accuracy of the recognition process. The next two sections will explain the two stages of our proposed approach. First, we will explain in the following section the steps in the identification of features from natural landmarks. After that, we will explain the steps of the second stage, which is the recognition of the natural landmarks using the identified features.

## 2. Stage 01: Natural Landmark Identification

The identification of features in natural landmarks can be divided into seven sub-steps. These steps are grouped into two groups: segmentation and filtering. First, we segment the captured scene using the three identified sub-steps: Narrow Down Filter, Estimation of Planes and Plane Edge Calculation. Later, we filter the segmented scene in order to generate a final cloud of edge points for object recognition.

**Figure 1:**
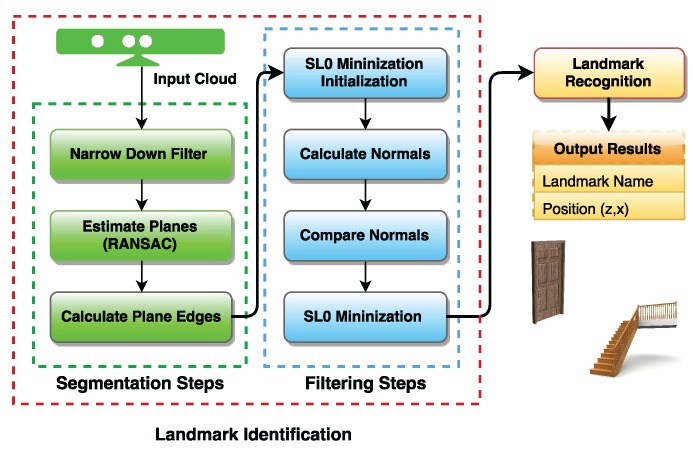
Natural landmark identification stage.

Figure 1 presents a diagram of the identification stage. The segmentation aims to pre-process the cloud resulting in a cloud containing only the points of interest. These points will then be treated and used in feature calculations. Afterwards, the resulting cloud is filtered in order to minimize the noise in the data. The final sub-steps in the filtering group perform calculations to estimate key characteristics from the observed landmarks.

### 2.1. Segmentation Sub-Steps

We use an RGB-D sensor (the Kinect) mounted on the mobile robot platform to acquire RGB and depth images from the surroundings. The Point Cloud Library (PCL) is used to transform raw RGB-D data ($X_{i}$, $Y_{i}$, R, G, B, D) to a point cloud $P_{i}=\left(X_{i},Y_{i},Z_{i},R_{i},G_{i},B_{i}\right)$, where i=1..N and *N* is the total number of points in the cloud. The point cloud is the chosen structure for data handling in our work. Note that a point is the most primitive geometric entity, thus allowing any geometric algorithm to be implemented over it. In fact, a Narrow Down Filtering (NDF) algorithm has to be applied for reducing the size of the point cloud, in order to allow real-time processing and decrease computational cost. We notice that structures such as walls, doors, staircases, pillars, and so on, have similar shapes from bottom up. Therefore, the filter is also used to minimize the size of the cloud, resulting in a target area of a horizontal rectangle, which, in turn, eliminates useless data present in the border of the field of view.

After the image acquisition, the NDF will prepare the image by returning points that have key characteristics (edge features) used in the landmark detection. The NDF, consequently, decreases the field of view of the robot to a slim horizontal rectangle with respect to the Kinect coordinate system [26]. The filter parameters can be seen in Table 1.

The following step is to identify the existing planes within the reduced field of view. The existing planes are present in almost any landmark. Usually, natural landmarks are corridors, water fountains, pillars, doors, staircases, and so on. By looking for these regular geometries (i.e., planes) in scene, we can find the predominant plane within the field of view. We use the RANSAC (RANdom SAmple Consensus) algorithm (available in the PCL) to estimate the predominant plane. This algorithm was modified in order to find the major planes in the rectangle box from the NDF. The major planes are characterized by having the highest number of inlier points.

**Figure 2:**
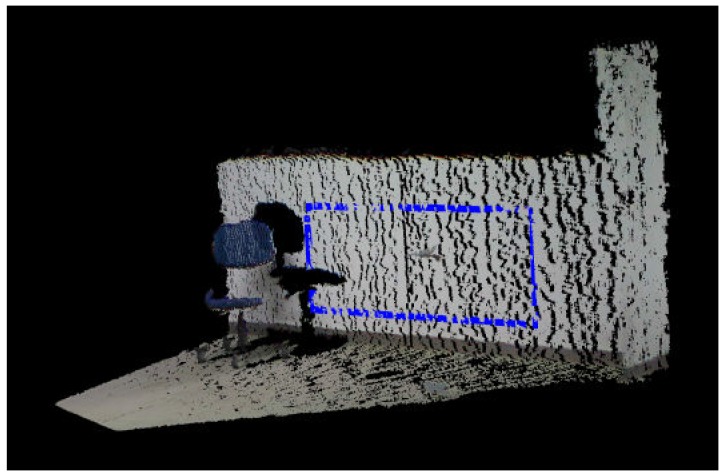
RANSAC and Narrow Down Filter result.

The modified plane estimation will exclude movable objects such as chairs, boxes, and so on, resulting in a cloud only with all features. This is perceptible in Figure 2 where there is a chair in the robot’s field of view. This object is then ignored after the NDF and the modified plane estimation steps. All detected planes are gathered in another cloud containing only estimated planes.

**Figure 3:**
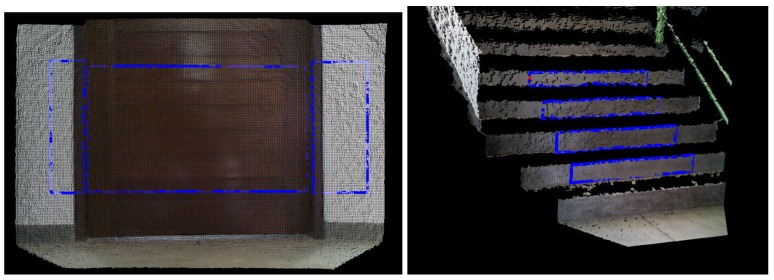
Images resulting from the segmentation step.

From the cloud of planes, we can start the detection of 3D edges. This procedure will minimize the number of used points. As a result, the edges can be computed quickly. The edges are found by using the Concave Hull algorithm, which is used to compute the envelope of a set of points in a given plane. From the given points, a non-convex polygon is generated that represents the area occupied by these points. However, as we can see in Figure 3, the segmentation step is not robust enough. The influence of robot orientation, object inclination and environment illumination can alter the result of the segmentation step. Therefore, planes with distortion and with outlying points are created as a result. Thus, we still need to pass the resulting cloud through another filtering process, the filtering sub-steps.

### 2.2. Filtering Sub-Steps

The filtering sub-steps are initiated using the $Sl_{0}$-norm algorithm. This algorithm will minimize the number of outliers, i.e., points that are not able to be classified as edge points but have been included in the resulting cloud from the previous step. This error in inclusion usually is due to environment disturbances or sensor noise. After the elimination of these outliers, the normal of each point in the cloud is calculated. These normals are then compared with the normals of previous points and the normals of following points. The points that do not have their normals parallel to the previous one or to the following one are classified as corner (edge) points. These edge points are gathered in a third cloud, called edge cloud. This new cloud is filtered once more through the $Sl_{0}$-norm algorithm, which will minimize the standard deviation of the angles between normal. Each aforementioned part of the filtering step will be described below.

The filter starts by traveling through each point of the resulting cloud and computing its normal. We assume that each detected point is on the surface of the observed object. Therefore, the normal of each point is orthogonal to the detected plane. The normal calculation is based on the work of Hoppe et al. [27]. Note that the influence of sensor noise and illumination variation has a direct impact on RGB-D sensor data acquisition. Thus, we need to filter the resulting cloud once more minimizing the number of outlier points in the cloud. When the curvature angle of a point (the angle between the normal vector and the surface plane) is different from approximately $90^{{}^{\circ}}$ (with a threshold sigma_min=0.01), then it is characterized as an outlier. The band-pass filter diminishes the number of points that will be candidates as edge points in the next sub-step.

After diminishing the amount of points, a comparison between normals of each pair of neighbor points is performed. Then, the difference angle (θ) between each pair of normal is calculated. In a region containing edge points, there is a high variance in direction between a pair of normal. When the calculated angle θ is bigger then $45^{{}^{\circ}}$, the points from the normals are considered edge points. The resulting points, which are considered edge points ($Pe_{i}$), usually lie in a wall corner.

This procedure is not fail-proof. Once again, the sensor noise can influence the selection of edge point with false-positives. For this reason, the following algorithm is used to minimize the presence of non-edge points by considering edge points the resulting vector of maximized zero components.

This sub-step filters the resulting keypoints using the smooth l0-norm minimization algorithm [28]. Following this approach, we adopt the $l_{0}$ norm of a non-zero components vector s, where $s=[s_{1},...,s_{N}]$ and s is the vector containing the calculated angles and its respective point in the edge cloud and $s_{i}=\theta_{i-1}^{i}$. To compute this, we assume that:

(1) $$\nu \left(s_{i}\right)=\left\{\begin{array}{cc} 0, & 45^{{}^{\circ}}<s_{i}<90^{{}^{\circ}},\\ 1, & otherwise. \end{array}\right.$$

Therefore, the $l_{0}$ norm of s is as follows:

(2) $${∥s∥}_{0}=\sum_{i=1}^{N}\nu \left(s_{i}\right).$$

The function ν is discontinuous. Thus, the norm is also discontinuous. From this analysis, we can per pass this issue by performing a smooth approximation of $\nu (s_{i})$. To this end we used a differentiable zero-mean Gaussian function as follows:

(3) $$f_{\sigma }\left(s\right)=exp\left(\frac{-s^{2}}{2\sigma^{2}}\right),$$

we have:

(4) $$\lim_{\sigma \to 0}f_{\sigma }\left(s\right)=\left\{\begin{array}{cc} 0, & s\neq 0,\\ 1, & s=0. \end{array}\right.$$

Consequently, $\lim_{\sigma \to 0}f_{\sigma }\left(s\right)=1-\nu \left(s\right)$, and, therefore, by defining:

(5) $$F_{\sigma }\left(s\right)=\sum_{i=1}^{N}f_{\sigma }\left(s_{i}\right),$$

we can have

(6) $$\lim_{\sigma \to 0}F_{\sigma }\left(s\right)=N-{∥s∥}_{0}.$$

and we take $\left[N-F_{\sigma }\left(s\right)\right]$ as an approximation of the ${∥s∥}_{0}$:

(7) $${∥s∥}_{0}\approx N-F_{\sigma }\left(s\right),$$

where σ is a trade-off between the accuracy and the smoothness of the approximation.

Equation (7) demonstrates the equivalence between the minimization of ${∥s∥}_{0}$ norm and the maximization of $F_{\sigma }\left(s\right)$. This is only possible for a small σ. Therefore, the *Normal Problem Equation* can be written as x=A*W, where *W* is a weight vector, *A* is the measurements matrix and the pseudo-inverse of *A* is $A_{pinv}$. From the pseudo-inverse, we calculate the initial value of *W* as $W=A_{pinv}*x$.

Thus, we perform the multiplication for each cell of the vector of the initial value of *W* by a weighted fraction of an approximation of its $l_{0}$-norm (σ). The approximation σ is the standard deviation of a zero-mean Gaussian function. Hence, we perform the subtraction of the residual from *W*. This is found through the error projection on $A_{pinv}$. The process is repeated until a threshold is achieved and the decreased value of σ reaches the reference Σ (see Algorithm 1).

**Figure 4:**
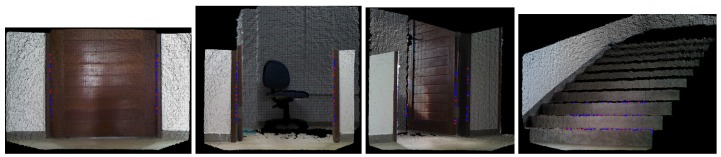
Results from the filtering sub-steps. Here, four cases of observation can be seen: closed door (far left), an open door (middle left), an open door with a vision of $45^{{}^{\circ}}$ (middle right) and a staircase with a vision of $45^{{}^{\circ}}$ (far right).

**Algorithm 1.** Smooth $l_{0}$-norm Algorithm**INPUT Data**: W = SL0(A,x,$\Sigma_{min}$,$A_{pinv}$);**OUTPUT Result**: return weight vector *W*;
1:$W=A_{pinv}*x$;2:σ=2*max(abs(W));3:$\mu_{0}=2$;4:L=3;5:$\sigma_{decrease}=0.5$;6:$\Sigma_{min}=0.01$7:**While** ($\sigma <\Sigma_{min}$);8:**For** (i=1:L);9:$\delta =W.*exp(-abs\left(W\right).^{2}/\sigma^{2})$;10:$W=W-\mu_{0}*\delta$;11:$W=W-A_{pinv}*\left(A*W-x\right)$;12:**end for**;13:$\sigma =\sigma *\sigma_{decrease}$;14:**end while**;


The results after the filtering process are presented in Figure 4. The key points that results from the filtering sub-steps are demonstrated in the four sub-figures (doors and a staircase).

## 3. Stage 02: Natural Landmark Recognition

**Figure 5:**
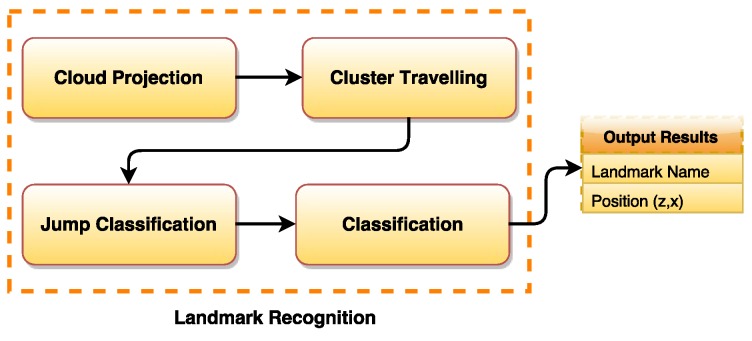
Natural landmark recognition stage.

The second phase of our approach is to recognize the natural landmarks in front of the robot. This is performed after separating the estimated key points. These points are now composing the edge point cloud. From these points, we will identify and recognize natural landmarks such as doors and staircases. Note that these types of natural landmarks are commonly seen in an indoor environment (e.g., a faculty building). These types of natural landmarks are not well addressed in literature regarding natural landmark recognition applied to mobile robot localization. To perform the landmark recognition, we use the depth and geometric information from the cloud that was processed in the identification stage. Similarly, the recognition stage can also be seen as a block diagram divided in four steps as shown in Figure 5.

The method applied here consists of filtering the resulting cloud in order to retrieve the intrinsic geometric characteristics regarding doors and stairs. First, the projection of the cloud on the plane XY is performed (floor plane), and several clusters of points are created. All the points of the cloud are then computed iteratively, and their spatial information (position and normal orientation) is extracted. The spatial information of the points are used to classify them as belonging to a certain landmark type. The doors or stairs characteristics are acquired when the difference between points is observed. We call this behavior a cluster “jump”. From these characteristics, the algorithm classifies the jumps. It is able to differentiate between stairs or doors jumps. Therefore, the proposed recognition part has the following four steps:

- **Step (1)** Project cloud on the floor plane;
- **Step (2)** Travel clusters (Jumps);
- **Step (3)** Classify jumps (Stairs or Doors);
- **Step (4)** Return the most appealing object (Final Recognition).

### 3.1. Cloud Projection

**Figure 6:**
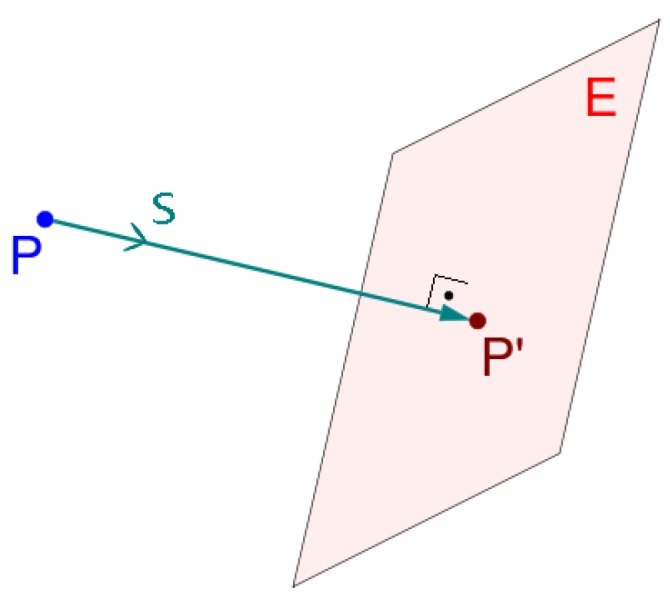
Orthogonal projection.

The projection of the resulting cloud from the identification step follows a criteria that states that a given cloud point $P(x^{\prime },y^{\prime },z^{\prime })$ and its projection $P^{\prime }$ determine a line in which the direction vector *s* coincides with the normal vector *N* of the projection plane *E* (Figure 6). The projection of point *P* into $p^{\prime }$ follows the Matrix:

$$\left[\begin{array}{c} x^{\prime }\\ y^{\prime }\\ z^{\prime }\\ 1 \end{array}\right]=\left[\begin{array}{cccc} 1 & 0 & 0 & 0\\ 0 & 0 & 0 & 0\\ 0 & 0 & 1 & 0\\ 0 & 0 & 0 & 1 \end{array}\right]\cdot \left[\begin{array}{c} x\\ y\\ z\\ 1 \end{array}\right].$$

**Figure 7:**
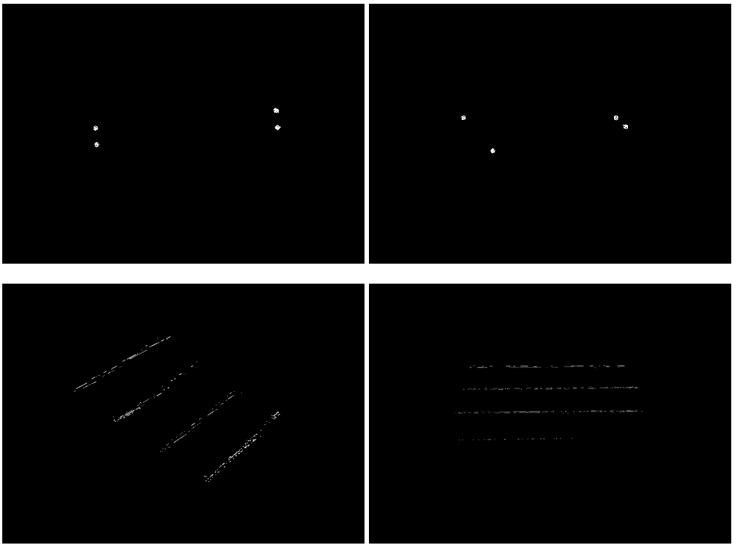
Images resulting from the projection step. Both top figures are clusters from the detection of doers and both bottom figures are from stairs.

Note that all points of the cloud are projected in the XZ plane, that is, by the sensor definition, a plane that is parallel to the ground if the sensor is in the upright position. For natural landmarks such as doors, it is observed that the resulting cloud after the projection forms a region with grouped points (Figure 7 top). However, in the stairs landmarks, the resulting cloud is a set of lines (Figure 7 bottom). These groups of points and lines are clusters of doors and clusters of stairs, respectively.

### 3.2. Cluster Traveling

The points are gathered nearby well defined clusters in the resulting projections from doors, similar to dots. This is the opposite in the staircase clusters that are well defined lines. After the projection, the cloud is submitted to an iterative procedure in which each point is visited and its spatial disposition is stored. After an empirically defined number of 15 points are visited, each consecutive point is analyzed and compared to the previous and to the next ones. This process is repeated until the last point is analyzed. When the difference between consecutive points matches a certain threshold, it is stated that the iteration has left the cluster, and the current point belongs to another cluster. This is called a cluster jump.

### 3.3. Jump Classification and Final Recognition

As mentioned above, a feature classification is achieved when a jump happens. Each cluster should be traveled and classified accordingly. For example, just before the jump, the point $P_{1}$($x_{1}$, $z_{1}$) is submitted to a comparison with a previous point $P_{0}$($x_{0}$, $z_{0}$) and to the next point $P_{2}$($x_{2}$, $z_{2}$). If it is observed that the previous point $P_{0}$ is similar to the current point $P_{1}$ and the values of the next point $P_{2}$ have increased, the jump is classified as a door jump. This is true because points belonging to door clusters are concentrated in a confined space. Stair jumps are classified when $P_{0}$ is much smaller than $P_{1}$, $P_{2}$ is greater than $P_{1}$ and there is a large variation in the depth(z) coordinate of $P_{2}$ from $P_{1}$, suggesting $P_{2}$ belongs to another cluster (another line in Figure 7 bottom).

## 4. Experiments and Results

**Figure 8:**
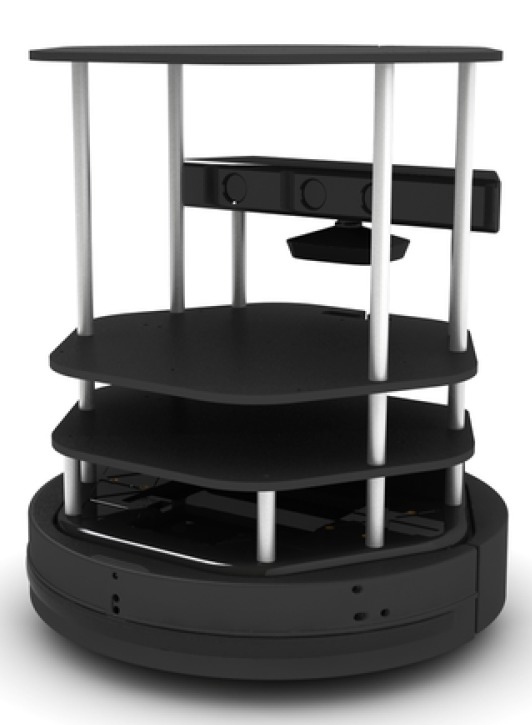
Turtlebot robot.

To verify and validate our approach, a total of eight experiments were performed to demonstrate their success. For the experiments, the robotic platform used was the TurtleBot 2 robot, which has a Microsoft Kinect 1 (Figure 8), and a Gyroscope and wheel odometry from the base of the robot, as capture sensors. The base and the Kinect were connected to a notebook, 86 GHz and 4 GB RAM. As software, the ROS framework was used in Ubuntu 14.04. We did not use any other type of sensor.

We use doors and staircases as natural landmarks in the experiments. We performed tests with different states of doors (open door, closed door and half closed door) and staircases with different widths. The difference between the semi-open state and open state is that, in the first, the door is not fully opened; in the second, the door is completely opened. All doors and staircases are from the same kind of environment (university buildings). However, in the considered environments, there are modern and older buildings, which, besides obeying a standard, produced little differences in the elements. Doors and stairs follow a standard of construction all around the world (in the geometrical sense). Color, texture and material can be different; however, the main features that are used by the algorithm are still present everywhere. For example, the measures of a door ranges from 0.60 m to 1.15 m in width, from 2.10 m to 2.20 m in height and between 0.10 m up to 0.15 m in depth. In the case of stairs, they should be some 0.2 m high and 0.3 m deep (the width does not matter in this case). More than that causes problems to the building users. This standard in construction enables us to look for this same pattern in the landmark observation, as we can see in Figure 7. Therefore, our algorithm is robust to color, texture and material variation. The only two exceptions for doors are double doors and sliding doors, which will be treated in future works due to their difference in the geometry. As we use the geometry of the landmark to identify it, we do not need an offline training step to recognize the natural landmark.

**Figure 9:**
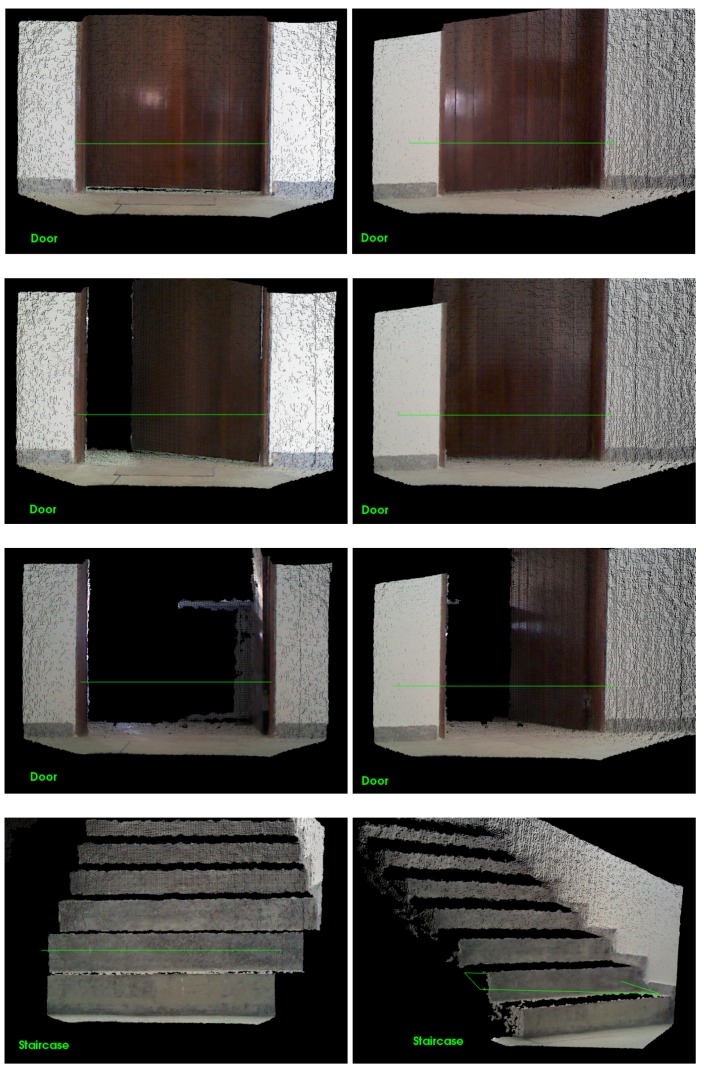
Recognition results. From top to bottom, left to right we see the detection of a closed door in 0 degree angle, a closed door in 45 degree angle, a partially closed door in 0 degree angle, a partially closed door in 45 degree angle, an open door in 0 degree angle, an open door in 45 degree angle, stairs in 0 degree angle and stairs in 45 degree angle.

This experiment uses 5000 (thousands) samples. These were taken in series during the algorithm running and with the robot in front of the landmark (varying the angle of observation). Each sample is a captured frame. We identify and recognize natural landmarks in each frame according to the features presented in an online fashion. The tests were performed with $0^{{}^{\circ}}$ and $45^{{}^{\circ}}$ viewing angles. The approach was successful in detecting both doors and stairs, having a very low index w.r.t. false positives and unidentified cases, as shown below. Figure 9 displays the detection data of all features at both angles.

In Table 2, one can observe the confusion matrices at angles $0^{{}^{\circ}}$ and a $45^{{}^{\circ}}$. Our approach had a 98.9% average of successful cases of natural landmarks (doors and stairs) recognition at a $0^{{}^{\circ}}$ viewing angle. By changing the viewing angle to $45^{{}^{\circ}}$, our approach decreased the rate of success in 0.86%. We performed an additional analysis, in which we found that the majority of false positives are due to noise from the Kinect sensor or erroneously identified planes. Furthermore, we present the distances (real and measured) between the robot and the landmark centroid in Table 3. The distances are measured in the *z*- (depth) and *x*- (width) axis of the Kinect sensor.

Table 4 presents a maximum mean absolute error for the half-open door with a $45^{{}^{\circ}}$ viewing angle in the *z*-axis of 0.018 m. This error was due to the empty space in front of the sensor that increases the difficulty in measurement by the Kinect 1.0 depth sensor. The maximum mean absolute error increased in the *x*-axis with a value less than 0.09 m in both the ladder and in the closed door cases with a $45^{{}^{\circ}}$ viewing angle. This table also presents the precision values of all four landmarks in each measured viewing angle ($0^{{}^{\circ}}$ and $45^{{}^{\circ}}$). The lower precision rate is in the ladder case with and observation angle of $45^{{}^{\circ}}$, which had a precision rate of 96.5%.

When analyzing the accuracy results of our approach through Table 5, we can state that it was satisfactory. Our approach had a 98.9% as the lower accuracy rate, again in the stair recognition cases with an observation angle of $45^{{}^{\circ}}$. We also chose a state-of-art approach to object detection and recognition that has similar characteristics with our proposed work in order to compare our results. The similarities of the chosen algorithm with our work are w.r.t. real-time processing, objects detection and recognition, false positive treatment and result accuracy. With this in perspective, the YOLO (You Only Look Once) [29] approach was used, which is characterized by being a deep neural network for objects detection and recognition in real time. The authors make use of a neural network capable of predicting and probabilistically affirming what object is referred to, after analyzing the image, besides processing in real time in a range of 45 frames per second. The YOLO approach was applied within the scope of identifying the objects treated in this work: doors and stairs. It is necessary to also note that such a comparison was only designed to analyze the accuracy results that our algorithm possesses in landmark recognition, since the YOLO model is a deep neural network not designed for mobile robotics, as our case is. That is, it has a longer processing time in addition to requiring previous training. We used the same database captured from the 5000 frames to test both approaches w.r.t. accuracy, and we only used the YOLO database to train the neural network. Taking this into account, our algorithm obtained a considerable result when compared to YOLO results.

The decreased values in both Precision and Accuracy in the staircase feature is due to the fact that some points in the stair cluster may be grouped when the projection forces two edges of staircase steps together, so the algorithm may conclude that there are a few door clusters. Notice that filtering techniques can be applied to minimize this situation and guarantee better results. Also taking into account the sensor-related deficiencies that are beyond the scope of this research, there are some situations that cannot be addressed by this algorithm. We can classify these situations into two main groups: environmental deficiencies and deficiencies related to the algorithm. Environmental deficiencies are caused by the incidence of light or by occlusions. It was possible to see, in the semi-open door case, that the algorithm is robust w.r.t. occlusion. However, ambient lighting is still a problem to be solved. This can be minimized if the RGB-D sensor is improved. In future works, we aim to change it to a Stereo RGB-D Zed Camera [30]. The second group is related to the algorithm, in which they derive from the descriptors that we use in our approach. Line projections can sometimes be confused, in the cases of a double door or a corridor being as straight as a closed door, which will also be corrected in future works.

## 5. Conclusions

This paper has proposed a robust for detecting natural landmarks using 3D point cloud data. The intention of this work was to propose a landmark recognition approach to be later used in localization. In dynamic unstructured environments such as industries, offices, faculties, and so on, mobile robots will depend on 3D data to localize themselves. Furthermore, with the pose of the detected objects, and thus with a natural landmark localization approach, a mobile robot can customize its trajectories with more efficiency. Therefore, we present here a generic approach to identify and recognize natural landmarks, based on the treatment of clustering process and application of noise minimization. The generalization is with respect to the robot pose in relation to the natural landmark. Height and depth information are extremely useful to mobile robots.

Our approach is performed online without the need of an offline training step preceding the landmark recognition. Furthermore, as our approach is based on landmark geometry, it also works with pillars, hallways, walls and so on, given the proper configuration. The performed experiments demonstrate that, when the robot is in front of the landmark, our approach has a 100% success in the classification of doorways (independent of the state of the door) and a 95.8% success in the classification of staircases. When the robot is in a $45^{{}^{\circ}}$ orientation with respect to the landmark our approach achieves an average of 99.74% success in classifying doorways and a 92.94% success in classifying staircases.

The case where two or more doors are close to one another separated by a pillar or a small wall represented a drawback in our approach. Cases such as this are usually interpreted as the wall being a closed door and the doors being the wall plane. We will approach this problem in future works by including these cases in the identification stage of the algorithm. Future works will also use this approach in the localization based on natural landmarks. This new localization approach is being currently researched to consider state-of-the-art advances in object recognition and visual odometry, and by considering other objects such as trash cans, water fountains, fixed tables, and so on. Finally, we aim to apply the recognition of natural landmarks to SLAM (Simultaneous Localization and Mapping) algorithms, where marks can be used to handle problems such as the kidnapped robot, border mapping, loop closure detection and relocation.

## Figures and Tables

**Table 1:** Narrow Down Filter parameters.

Axis	Lower Values	Higher Values
*y*	−0.6 m	0.2 m
*x*	−0.6 m	0.6 m

**Table 2:** Recognition confusion matrices.

**$0^{{}^{\circ}}$ Measurement**				
**Landmark**	**Predicted**			
	**Open** **Door**	**Semi-Open** **Door**	**Closed** **Door**	**Stairs**
**Open Door**	5000	0	0	0
**Semi-Open Door**	0	5000	0	0
**Closed Door**	0	0	5000	0
**Stairs**	0	0	161	4791
**$45^{{}^{\circ}}$ Measurement**				
**Landmark**	**Predicted**			
	**Open** **Door**	**Semi-Open** **Door**	**Closed** **Door**	**Stairs**
**Open Door**	4983	0	0	17
**Semi-Open Door**	0	4979	0	21
**Closed Door**	0	0	4999	1
**Stairs**	0	0	167	4647

**Table 3:** Mean measured distances (in meter).

Features	Angle	Real Cen (*z*)	Real Cent (*x*)	Measured Cen (*z*)	Measured Cen (*x*)
**Open Door**	0	1.4	0	1.41	0.004
**H-Open Door**	0	1.8	0	1.81	0.004
**Closed Door**	0	1.5	0	1.49	0.008
**Staircase**	0	1.2	0	1.191	0.022
**Open Door**	45	1.6	0	1.583	0.014
**H-Open Door**	45	1.5	0	1.482	0.04
**Closed Door**	45	1.5	0	1.493	0.021
**Staircase**	45	1.3	0	1.28	0.007

**Table 4:** Absolute error and precision.

Features	Angle (°)	Precision (%)	Absolute Error *z* (m)	Absolute Error *x* (m)
**Open Door**	0	100	0.01	0.004
**Semi-Open Door**	0	100	0.01	0.051
**Closed Door**	0	100	0.01	0.018
**Stairs**	0	96.7	0.009	0.078
**Open Door**	45	99.6	0.017	0.066
**Semi-Open Door**	45	99.5	0.018	0.024
**Closed Door**	45	99.9	0.007	0.086
**Stairs**	45	96.5	0.02	0.086

**Table 5:** Comparison accuracy values with YOLO (You Only Look Once).

**$0^{{}^{\circ}}$ Measurement**		
**Landmarks**	**YOLO %**	**Our Approach %**
**Open Door**	95.3	100
**Semi-Open Door**	97.0	100
**Closed Door**	100	100
**Stairs**	94.57	99.1
**$45^{{}^{\circ}}$ Measurement**		
**Landmarks**	**YOLO %**	**Our Approach %**
**Open Door**	93.6	99.0
**Semi-Open Door**	94.47	99.0
**Closed Door**	99.9	99.1
**Stairs**	95.5	98.9
